# Risk of osteoporosis and pathologic fractures in cancer patients who underwent hematopoietic stem cell transplantation: a nationwide retrospective cohort study

**DOI:** 10.18632/oncotarget.16746

**Published:** 2017-03-31

**Authors:** Jiun-Nong Lin, Hsuan-Ju Chen, Chih-Hui Yang, Chung-Hsu Lai, Hsi-Hsun Lin, Chao-Sung Chang, Ji-An Liang

**Affiliations:** ^1^ Department of Critical Care Medicine, E-Da Hospital, I-Shou University, Kaohsiung, Taiwan; ^2^ School of Medicine, College of Medicine, I-Shou University, Kaohsiung, Taiwan; ^3^ Division of Infectious Diseases, Department of Internal Medicine, E-Da Hospital, I-Shou University, Kaohsiung, Taiwan; ^4^ Management Office for Health Data, China Medical University Hospital, Taichung, Taiwan; ^5^ College of Medicine, China Medical University, Taichung, Taiwan; ^6^ Department of Biological Science and Technology, Meiho University, Pingtung, Taiwan; ^7^ Department of Hematology/Oncology, E-Da Cancer Hospital, Kaohsiung, Taiwan; ^8^ Graduate Institute of Clinical Medical Science and School of Medicine, College of Medicine, China Medical University, Taichung, Taiwan; ^9^ Department of Radiation Oncology, China Medical University Hospital, Taichung, Taiwan

**Keywords:** hematopoietic stem cell transplantation, osteoporosis, fracture, bone loss

## Abstract

**Background:**

Long-term data on post-hematopoietic stem cell transplantation (HSCT) osteoporosis and fracture are limited. This study evaluated the long-term risk of osteoporosis and fracture in cancer patients who underwent HSCT.

**Results:**

The incidence density rate of osteoporosis was 12.5 per 1000 person-years in the HSCT group, which was significantly higher than that in the non-HSCT group (5.65 per 1000 person-years) after adjustment for associated factors and consideration of competing risk factors (adjusted subhazard ratio, 1.48; 95% confidence interval, 1.06–2.07). The incidence density rate of fracture was 4.89 per 1000 person-years in the HSCT group, and the risk of fracture was 1.40 times higher in the HSCT group than in the non-HSCT group (95% confidence interval, 0.83–2.40). The vertebra was the most common site of fracture after HSCT (68.4%). The risk of osteoporosis and fracture significantly increased in post-HSCT patients with both hematological malignancies and solid tumors. Both autologous and allogeneic HSCTs increased the risk of osteoporosis, whereas only autologous HSCT recipients had an increased risk of fracture.

**Materials and Methods:**

This nationwide retrospective cohort study analyzed data from Taiwan's National Health Insurance Research Database. We identified an HSCT group comprising 1040 cancer patients who underwent HSCT during 2000–2008 and a non-HSCT group comprising 4160 propensity score-matched cancer patients who did not undergo HSCT. All patients were followed up until the occurrence of osteoporosis; fracture; December 31, 2011; or withdrawal from the insurance program.

**Conclusions:**

HSCT recipients have an increased risk of osteoporosis.

## INTRODUCTION

Hematopoietic stem cell transplantation (HSCT) involves the intravenous infusion of autologous or allogeneic multipotent hematopoietic stem cells for re-establishing recipients’ hematopoietic and immunological functions [1, 2]. HSCT is primarily recommended for patients with hematopoietic diseases and malignancies, and it has been increasingly used worldwide in the past three decades [1, 2].

Despite substantial progress in the HSCT technique and the subsequent care, many complications including mucositis, acute and chronic graft-versus-host disease, infections, hepatic veno-occlusive disease, lung injury, and secondary cancer are prevalent after the transplantation [2]. Bone loss is a common complication following HSCT [3–7]. The pathogenesis of osteoporosis and the subsequent fracture involves multiple factors including the transplant procedure, secondary hypogonadism, chemotherapy, radiotherapy, immunosuppressive therapies, reduced mobility and growth factors, and abnormal metabolism and absorption of calcium and vitamin D because of kidney, liver, and bowel dysfunctions [3, 8].

Several studies have reported bone loss after HSCT [9–15]. In contrast to data on the short-term effects of bone loss, data on the long-term effects of bone loss after HSCT are limited [7, 9, 13–15]. In Taiwan, HSCT has been a common therapy for hematopoietic diseases and malignancies since 1983 [16]. Approximately 200 transplantations have been performed annually in recent years, and more than 2200 patients have undergone HSCT until 2008 [16]. The present study analyzed the incidence and risk of osteoporosis and fracture in cancer patients who underwent HSCT by using Taiwan's National Health Insurance Research Database (NHIRD).

## RESULTS

This study included 1040 cancer patients who underwent HSCT (HSCT group) and 4160 cancer patients who did not undergo HSCT (non-HSCT group; Table 1). The mean age of patients in the HSCT group was 34.8 years (standard deviation, 16.0 years); this group had male predominance (60.8%). After propensity score matching, both the HSCT and non-HSCT groups had similar sex, age, comorbidities, and cancer types (all standardized differences < 0.1).

**Table 1:** Demographic factors and comorbidities of enrolled patients in this study

	Non-HSCT group (*N* = 4160)		HSCT group (*N* = 1040)		Standardized difference
	*n*	%	*n*	%	
**Sex**					< 0.001
Women	1633	39.3	408	39.2	
Men	2527	60.7	632	60.8	
**Age of HSCT (mean ± standard deviation) (year)**	36.3 ± 20.5		34.8 ± 16.0		0.08
**Comorbidity**					
Diabetes mellitus	195	4.69	61	5.87	0.05
Hyperlipidemia	478	11.5	116	11.2	0.01
Hypertension	430	10.3	141	13.6	0.09
Coronary artery disease	221	5.31	69	6.63	0.06
Depression	159	3.82	41	3.94	0.006
Stroke	50	1.20	16	1.54	0.03
Chronic obstructive pulmonary disease	301	7.24	80	7.69	0.02
Chronic kidney disease	288	6.92	81	7.79	0.03
**Cancer**					
Head and neck cancer	200	4.81	50	4.81	< 0.001
Digestive system cancers	24	0.58	6	0.58	< 0.001
Lung and mediastinum cancers	44	1.06	11	1.06	< 0.001
Bone and soft-tissue cancers	40	0.96	10	0.96	< 0.001
Breast cancer	28	0.67	7	0.67	< 0.001
Genitourinary tract cancers	68	1.63	17	1.63	< 0.001
Hematologic malignancies	3696	88.8	924	88.8	< 0.001
Others	60	1.44	15	1.44	< 0.001

HSCT: hematopoietic stem cell transplant.

During the follow-up period, 47 and 125 patients in the HSCT and non-HSCT groups developed osteoporosis, respectively (Table 2). The incidence density rates of osteoporosis were 12.5 and 5.65 per 1000 person-years in the HSCT and non-HSCT groups, respectively. Multivariate Cox proportional regression analysis indicated a significantly higher risk of osteoporosis in the HSCT group than in the non-HSCT group after adjustment for sex, age, comorbidity, and cancer type (adjusted hazard ratio [HR], 2.53; 95% confidence interval [CI], 1.79–3.57). After consideration of the competing risk factor for death, the corresponding subhazard ratio (SHR) of osteoporosis was 1.48 (95% CI, 1.06–2.07) in the HSCT group compared with the non-HSCT group.

**Table 2:** Incidence density rates and hazard ratios of osteoporosis and fracture in the HSCT group comparing with the non-HSCT group

	Non-HSCT group			HSCT group			HR (95% CI)		SHR (95% CI)	
	Event no.	Person-years	IR	Event no.	Person-years	IR	Crude	Adjusted^a^	Crude	Adjusted^a^
Osteoporosis	125	22,114	5.65	47	3775	12.5	2.14 (1.53–3.00)***	2.53 (1.79–3.57)***	1.54 (1.09–2.13)*	1.48 (1.06–2.07)*
Fracture	54	22,383	2.41	19	3889	4.89	1.97 (1.17–3.33)*	2.28 (1.34–3.89)**	1.42 (0.84–2.40)	1.40 (0.83–2.40)

HSCT: hematopoietic stem cell transplant; IR: incidence density rate per 1000 person-years; HR: hazard ratio; CI: confidence interval; SHR: subhazard ratio.

^a^Model adjusting for sex, age, diabetes mellitus, hyperlipidemia, hypertension, coronary artery disease, depression, stroke, chronic obstructive pulmonary disease, chronic kidney disease, and the type of cancer.

*P < 0.05, **P < 0.01, ***P < 0.001.

The sex-stratified analysis demonstrated that women had a higher incidence of osteoporosis than did men in both the HSCT and non-HSCT groups (Table 3). However, women and men had a similar increased risk of osteoporosis in the HSCT and non-HSCT groups (adjusted HR, 2.59; 95% CI, 1.64–4.09 in women; adjusted HR, 2.58; 95% CI, 1.51–4.41 in men). The age-specific analysis indicated that patients who underwent HSCT had a higher risk of osteoporosis than did those who did not undergo HSCT in the age groups of < 18 years (adjusted HR, 5.33; 95% CI, 1.70–16.7) and 18–50 years (adjusted HR, 3.45; 95% CI, 2.15–5.55). Irrespective of comorbidities, the HSCT group had a higher risk of osteoporosis than did the non-HSCT group (adjusted HR, 2.92; 95% CI, 1.84–4.63 in patients without comorbidity; adjusted HR, 2.02; 95% CI, 1.19–3.42 in patient with comorbidity). The risk of osteoporosis was significantly higher in patients with hematological malignancies (adjusted HR, 2.32; 95% CI, 1.59–3.40) and solid tumors (adjusted HR, 5.92; 95% CI, 2.34–15.0) in the HSCT group than in those in the non-HSCT group. A significant interaction was observed between HSCT and age on the occurrence of osteoporosis (*P* = 0.003). However, the interaction was not significant between HSCT and sex (*P* = 0.91), HSCT and comorbidity (*P* = 0.12), and HSCT and cancer type (*P* = 0.10). Furthermore, we examined the interaction between factors in the model developed for osteoporosis. No significant interactions were identified between age and sex (*P* = 0.18), transplant type and cancer type (*P* = 0.58), and comorbidity and age (*P* = 0.47).

**Table 3:** Incidence density rates and hazard ratios of osteoporosis and fracture in patients with and without HSCT stratified by sex, age, comorbidity, and underlying cancers

	Non-HSCT group			HSCT group			HR (95% CI)		*P* for interaction
	Event no.	Person-years	IR	Event no.	Person-years	IR	Crude	Adjusteda	
**Osteoporosis**									
**Sex**									0.91
Women	74	8915	8.30	27	1500	18.0	2.10 (1.35–3.27)**	2.59 (1.64–4.09)***	
Men	51	13,199	3.86	20	2275	8.79	2.21 (1.31–3.70)**	2.58 (1.51–4.41)***	
**Age of HSCT (year)**									0.003
< 18	8	6307	1.27	5	576	8.68	5.69 (1.84–17.5)**	5.33 (1.70–16.7)**	
18–50	40	11162	3.58	31	2616	11.9	3.33 (2.08–5.32)***	3.45 (2.15–5.55)***	
> 50	77	4646	16.6	11	582	18.9	1.10 (0.59–2.06)	1.10 (0.58–2.10)	
**Comorbidity**^†^									0.12
No	58	16,854	3.44	27	2601	10.4	2.94 (1.86–4.64)***	2.92 (1.84–4.63)***	
Yes	67	5260	12.7	20	1173	17.1	1.34 (0.82–2.22)	2.02 (1.19–3.42)**	
**Type of cancer**									0.10
Hematologic malignancy	115	19824	5.80	38	3407	11.2	1.86 (1.29–2.68)**	2.32 (1.59–3.40)***	
Solid tumor	10	2290	4.37	9	368	24.5	5.85 (2.36–14.5)***	5.92 (2.34–15.0)***	
**Fracture**									
**Sex**									0.13
Women	28	9107	3.07	6	1596	3.76	1.21 (0.50–2.92)	1.51 (0.61–3.74)	
Men	26	13,276	1.96	13	2293	5.67	2.78 (1.43–5.43)**	3.21 (1.62–6.37)***	
**Age of HSCT (year)**									0.62
< 18	5	6313	0.79	2	5873	3.41	3.47 (0.66–18.2)	3.41 (0.63–18.5)	
18–50	19	11259	1.69	11	2707	4.06	2.46 (1.17–5.17)*	2.68 (1.26–5.72)*	
> 50	30	4811	6.24	6	595	10.1	1.53 (0.64–3.67)	1.64 (0.67–4.02)	
**Comorbidity**^b^									0.79
No	28	16,983	1.65	10	2687	3.72	2.34 (1.09–4.61)*	2.26 (1.09–4.69)*	
Yes	26	5400	4.81	9	1203	7.48	1.52 (0.71–3.25)	1.94 (0.88–4.28)	
**Type of cancer**									0.11
Hematologic malignancy	49	20087	2.44	14	3504	3.99	1.58 (0.87–2.87)	1.90 (1.03–3.50)*	
Solid tumor	5	2297	2.18	5	385	13.0	6.26 (1.80–21.7)**	5.79 (1.67–20.1)**	

HSCT: hematopoietic stem cell transplant; IR: incidence density rate per 1000 person-years; HR: hazard ratio; CI: confidence interval.

^a^Model mutually adjusting for sex, age, diabetes mellitus, hyperlipidemia, hypertension, coronary artery disease, depression, stroke, chronic obstructive pulmonary disease, chronic kidney disease, and the type of cancer.

^b^Including diabetes mellitus, hyperlipidemia, hypertension, coronary artery disease, depression, stroke, chronic obstructive pulmonary disease, and chronic kidney disease.

*P < 0.05, **P < 0.01, ***P < 0.001.

A total of 19 patients in the HSCT group and 54 patients in the non-HSCT group developed fractures (Table 2). All these patients with fracture had also received a diagnosis of osteoporosis. The incidence density rates of fracture in the HSCT and non-HSCT groups were 4.89 and 2.41 per 1000 person-years, respectively. Moreover, the multivariate Cox proportional regression analysis demonstrated a significantly higher risk of fracture in the HSCT group than in the non-HSCT group (adjusted HR, 2.28; 95% CI, 1.34–3.89). The corresponding SHR of fracture was 1.40 (95% CI, 0.83–2.40) in the HSCT group compared with the non-HSCT group after consideration of the competing risk factor for death. The sex-stratified analysis indicated that only men who underwent HSCT had a higher risk of fracture than did those who did not undergo HSCT (adjusted HR, 3.21; 95% CI, 1.62–6.37; Table 3). The age-specific analysis demonstrated that patients in the HSCT group had a higher risk of fracture than did those in the non-HSCT group in the age group of 18–50 years (adjusted HR, 2.68; 95% CI, 1.26–5.72). The comorbidity-specific analysis indicated that the risk of fracture in patients without any comorbidity was higher in the HSCT group than in the non-HSCT group (adjusted HR, 2.26; 95% CI, 1.09–4.69). Furthermore, the risk of fracture was significantly higher in patients with hematological malignancies (adjusted HR, 1.90; 95% CI, 1.03–3.50) and solid tumors (adjusted HR, 5.79; 95% CI, 1.67–20.1) in the HSCT group than in those in the non-HSCT group. The analysis of interaction between HSCT and each of the factors for fracture showed that sex (*P* = 0.13), age (*P* = 0.62), comorbidity (*P* = 0.79), and cancer type (*P* = 0.11) did not have significant interactions with HSCT. All the interactions between age and sex (*P* = 0.17), transplant type and cancer type (*P* = 0.52), and comorbidity and age (*P* = 0.16), were nonsignificant in the model developed for fracture.

The sites of fracture are listed in Table 4. Vertebral fracture accounted for 68.4% and 55.6% of fractures in the HSCT and non-HSCT groups, respectively. The risk of vertebral fracture was significantly higher in the HSCT group than in the non-HSCT group (adjusted HR, 2.73; 95% CI, 1.40–5.33). We analyzed the risk of osteoporosis and fracture according to the transplant types (Table 5). The risk of post-HSCT osteoporosis was high in both autologous HSCT (adjusted HR, 2.57; 95% CI, 1.66–3.99) and allogeneic HSCT (adjusted HR, 2.41; 95% CI, 1.51–3.87). The risk of fracture was significantly high in patients who underwent autologous HSCT (adjusted HR, 3.24; 95% CI, 1.78–5.90); however, no significantly increased risk of fracture was observed in patients following allogeneic HSCT (adjusted HR, 1.28; 95% CI, 0.51–3.22).

**Table 4:** Incidence density rates and hazard ratios for different subtypes of pathologic fracture in the HSCT group comparing with the non-HSCT group

Subtypes of fracture (ICD-9-CM code)	Non-HSCT group		HSCT group		HR (95% CI)	
	Event no.	IR	Event no.	IR	Crude	Adjusted^a^(95% CI)
Pathologic fracture (733.1)	4	0.18	0	0.00	-	-
Pathologic fracture, unspecified site (733.10)	4	0.18	1	0.26	1.43 (0.16–12.8)	1.72 (0.18–16.8)
Pathologic fracture of humerus (733.11)	4	0.18	2	0.51	2.83 (0.52–15.5)	3.42 (0.60–19.7)
Pathologic fracture of distal radius and ulna (733.12)	0	0.00	0	0.00	-	-
Pathologic fracture of vertebrae (733.13)	30	1.34	13	3.34	2.43 (1.27–4.67)**	2.73 (1.40–5.33)**
Pathologic fracture of neck of femur (733.14)	4	0.18	1	0.26	1.35 (0.15–12.2)	1.86 (0.20–17.6)
Pathologic fracture of other specified part of femur (733.15)	2	0.09	0	0.00	-	-
Pathologic fracture of tibia or fibula (733.16)	2	0.09	2	0.51	5.67 (0.79–40.5)	9.30 (0.97–89.4)
Pathologic fracture of other specified site (733.19)	4	0.18	0	0.00	-	-

HSCT: hematopoietic stem cell transplant; IR: incidence density rate per 1000 person-years; HR: hazard ratio; CI: confidence interval.

^a^Model adjusting for sex, age, diabetes mellitus, hyperlipidemia, hypertension, coronary artery disease, depression, stroke, chronic obstructive pulmonary disease, chronic kidney disease, and the type of cancer.

**P < 0.01.

**Table 5:** Incidence density rates and hazard ratios of osteoporosis and fracture in different types of transplants

Variable (ICD-9-CM procedure code)	*N*	Event no.	Person-years	IR	Adjusted HR^a^ (95% CI)
**Osteoporosis**					
**Non-HSCT group**	4160	125	22,114	5.65	1 (Reference)
**HSCT group (41.0)**					
Bone marrow transplant, not otherwise specified (41.00)	30	1	146	6.83	2.17 (0.30–15.7)
Autologous HSCT (41.01, 41.04, 41.07, 41.09)	428	25	1732	14.4	2.57 (1.66–3.99)***
Allogeneic HSCT (41.02, 41.03, 41.05, 41.08)	570	21	1871	11.2	2.41 (1.51–3.87)***
Cord blood stem cell transplant (41.06)	12	0	26	0	-
**Fracture**					
**Non-HSCT group**	4160	54	22,383	2.41	1 (Reference)
**HSCT group (41.0)**					
Bone marrow transplant, not otherwise specified (41.00)	30	0	154	0	-
Autologous HSCT (41.01, 41.04, 41.07, 41.09)	428	14	1763	7.94	3.24 (1.78–5.90)***
Allogeneic HSCT (41.02, 41.03, 41.05, 41.08)	570	5	1946	2.57	1.28 (0.51–3.22)
Cord blood stem cell transplant (41.06)	12	0	26	0	-

HSCT: hematopoietic stem cell transplant; IR: incidence density rate per 1000 person-years; HR: hazard ratio; CI: confidence interval.

^a^Model adjusting for sex, age, diabetes mellitus, hyperlipidemia, hypertension, coronary artery disease, depression, stroke, chronic obstructive pulmonary disease, chronic kidney disease, and the type of cancer.

***P < 0.001.

## DISCUSSION

In this large-scale, nationwide, retrospective cohort study, we investigated the risk of osteoporosis and fracture in cancer patients who underwent HSCT in Taiwan. According to our literature review, no large-scale study has evaluated the risk of osteoporosis and fracture in cancer patients who underwent HSCT in the Asian population.

In our study, the HSCT group had a 2.53 times higher risk of osteoporosis and a 2.28 times higher risk of fracture than did the non-HSCT group after adjustment for sex, age, comorbidity, and cancer type. After considering the competing risk factor for death, the risk of osteoporosis was significantly higher in the HSCT group (SHR, 1.48; 95% CI, 1.06–2.07); however, the effect of HSCT on fracture was not significant (SHR, 1.40; 95% CI, 0.83–2.40). Several studies have reported the effect of HSCT on both osteoporosis and fracture [9–15]. For example, a large-scale study by Pundole *et al*. [7] reported that HSCT recipients were approximately 7–9 times more likely to develop fracture than the general population in the United States of America. The nonsignificant effect of HSCT on fracture in the present study may be due to a small number of fractures.

Age at the time of HSCT has an effect on bone loss after this procedure [26, 27]. Bone mineral density (BMD) constantly increases during childhood and adolescence [28]. Petryk *et al*. [26] reported that patients who underwent HSCT at age < 10 years had a significantly lower BMD than did those who underwent HSCT at age > 18 years. HSCT and its related therapy may affect bone acquisition and damage bone cells and the stromal microenvironment during the critical period of bone development [26]. In our study, the incidence of osteoporosis increased with age at HSCT receipt. A significant interaction was observed between HSCT and age on osteoporosis (*P* = 0.003). The risk of osteoporosis after HSCT was higher in patients who underwent HSCT at age < 18 years (adjusted HR, 5.33) than in patients who underwent HSCT at the age of 18–50 years (adjusted HR, 3.45) and age > 50 years (adjusted HR, 1.10). Our results are consistent with those reported by Petryk *et al*. [26].

Our study results revealed that vertebral fracture was the leading site of fracture in the HSCT group (68.4%). Pundole *et al*. [7] also reported that vertebral fracture was the most common site of post-HSCT fracture (53%). Bone loss can occur at any site of the body after HSCT. In one study, BMD decreased by 0.6% in the spine, 0.4% in the total body, 2.3% in the femoral neck, and 3.5% in the Ward triangle every year [13]. In the general population, the hip, forearm, and spine are the most common sites of osteoporotic facture, and vertebral fracture accounts for approximately 15% of all fractures [29, 30]. The exact reason of the predominance of vertebral fracture after HSCT remains unknown. Additional studies are warranted to explore the cause of this result.

In the current study, the HSCT group had a higher risk of fracture than did the non-HSCT group irrespective of the presence of comorbidity. However, statistical significance was observed only in patients without comorbidity (adjusted HR, 2.26; 95% CI, 1.09–4.69) and not in those with comorbidity (adjusted HR, 1.94; 95% CI, 0.88–4.28). A small case number of fractures in the subgroup may be a potential bias. Another possible interpretation for this phenomenon is the secondary effects of the comorbidity-based treatment. For example, corticosteroid treatment in chronic obstructive pulmonary disease can result in bone loss [31].

Studies comparing the difference in the incidence and severity of bone loss and fractures between autologous and allogeneic HSCT recipients have yielded inconsistent results. Several studies have reported a higher reduction in BMD in allogeneic HSCT recipients than in autologous HSCT recipients [32–34]. However, Yao *et al*. [35] revealed similar incidence and severity in BMD reduction in autologous and allogeneic HSCT recipients. Pundole *et al*. [7] described an approximately two-fold higher risk of fracture in autologous HSCT recipients than in allogeneic HSCT recipients. Consistent with their results, our study results revealed a higher risk of osteoporosis in autologous than in allogeneic HSCT recipients. Regarding fracture, only autologous HSCT recipients demonstrated an increased risk of fracture. Although the incidence of fracture increased in allogeneic HSCT recipients compared with the non-HSCT group, it was not statistically significant. The dose and duration of immunosuppressants as well as the use and intensity of radiotherapy can vary among patients with HSCT. These factors could affect the occurrence of osteoporotic fracture and may have caused a bias in our study results [3, 35]. Moreover, the occurrence rate of graft-versus-host disease can affect the risk of osteoporosis. However, no accurate information was available about these factors in the database used in this study. Therefore, we cannot conclude the reasons for the difference among these studies.

The major strengths of this study include the large-scale nationwide design, comprehensive demographic data, and the long follow-up duration. However, the study has several limitations. First, the measurement of BMD through dual-energy X-ray absorptiometry is the gold standard for diagnosis of osteoporosis [36]. The NHIRD does not contain data on the BMD Z-scores or T-scores. Therefore, we could not confirm whether the diagnosis met BMD Z-scores or T-scores defined for osteoporosis. In the current study, the diagnoses of osteoporosis were based on the International Classification of Diseases, Ninth Revision, Clinical Modification (ICD-9-CM) codes, which were judged and determined by clinical physicians according to clinical standards. Moreover, a committee in the National Health Insurance comprising medical reimbursement specialists regularly reviews the charts and assesses the accuracy of these claims files. Inaccurate coding of diseases would result in no reimbursement, and the institutions would be subjected to fines. Therefore, the diagnoses and codes for osteoporosis used in this study should be correct and reliable. Second, information on several factors for osteoporosis and osteoporotic fractures, such as smoking, body mass index, calcium intake, diet supplement, physical activity, socioeconomic status, and some treatments, are unavailable in the NHIRD. These factors could have biased the study results. Third, some infections were significantly associated with osteoporosis and fracture. However, information on the laboratory confirmation of infections was unavailable in the NHIRD. Fourth, the HSCT population could have been followed up more frequently than the control group and hence might have a higher rate of osteoporosis diagnosis. This could attenuate the strength of the association between osteoporosis and HSCT. Finally, the statistical evidence derived from a retrospective study is generally weaker than that from randomized controlled trials.

In conclusion, our study results revealed a significant risk of osteoporosis following HSCT in cancer patients. Both osteoporosis and subsequent fracture largely affect the quality of life and increase morbidity and mortality. Thus, HSCT recipients should be considered at a high risk of osteoporosis, and regular follow-up and preventive measures against these complications are suggested for all HSCT recipients of cancer patients.

## MATERIALS AND METHODS

### Data source

Taiwan's National Health Insurance program, established in 1995, is a population-based mandatory health insurance program, covering approximately 99.9% of the Taiwanese population. In this study, we used datasets from the Registry for Catastrophic Illness Patient Database of the NHIRD. Under the National Health Insurance program, a catastrophic illness certificate is provided for patients with diseases such as cancer, end-stage renal disease, and organ transplantation. The Catastrophic Illness Patient Database is highly accurate because the certification is reviewed by relevant experts after scrupulous verification of medical records, imaging studies, and pathological findings. This database contains outpatient and inpatient information such as demographic data, visit dates, and ICD-9-CM codes of the diagnosis and procedures. For privacy reasons, all identifiable patient information in the NHIRD is encrypted before releasing it for research purposes.

### Ethics statement

The NHIRD encrypts patient personal information to protect privacy and provides researchers with anonymous identification numbers associated with relevant claims information. Therefore, patient consent is not required to access the NHIRD. This study was approved to fulfill the condition for exemption by the Institutional Review Board of China Medical University (CMUH104-REC2-115-CR1). The institutional review board also specifically waived the consent requirement.

### Study population and primary outcome

We enrolled patients of all ages who had cancer and had undergone HSCT (ICD-9-CM for procedure code 41.0) between January 1, 2000, and December 31, 2008. The date of transplantation was defined as the index date. For comparison, patients with cancer who did not receive HSCT during the same period were randomly selected and matched with the study cohort at a 4:1 ratio by propensity score. The common comorbidities that could be associated with osteoporosis or fracture were included for analysis, namely diabetes mellitus (ICD-9-CM 250) [17], hyperlipidemia (ICD-9-CM 272) [18], hypertension (ICD-9-CM 401–405) [19], coronary artery disease (ICD-9-CM 410–414) [19], depression (ICD-9-CM 296.2, 296.3, 300.4, 311) [20], stroke (ICD-9-CM 430–438) [21], chronic obstructive pulmonary disease (ICD-9-CM 491–493, 496) [22], and chronic kidney disease (ICD-9-CM 580–589) [23].

The propensity score matching, which is the logit (probability) for HSCT, was derived from a logistic regression model by using sex, age, year of the diagnosis of cancer, year of the index date, the aforementioned comorbidities, and cancer types (head and neck cancers [ICD-9-CM 140–149, 160, 161, 190–194], digestive system cancers [ICD-9-CM 150–157], lung and mediastinum cancers [ICD-9-CM 162–165], bone and soft-tissue cancers [ICD-9-CM 170–173], breast cancer [ICD-9-CM 174–175], genitourinary tract cancers [ICD-9-CM 179–189], hematologic malignancies [ICD-9-CM 200–208], and others [ICD-9-CM 158, 159, 176, 195–199]). The underlying cancers indicated for HSCT were classified into hematologic malignancies (ICD-9-CM 200–208) and solid tumors (ICD-9-CM 140–199). Patients with a history of osteoporosis and fracture and those with incomplete age or sex information in the database were excluded. In addition, to avoid fractures secondary to metastatic lesions, patients with bone metastases (ICD-9-CM 198.5) were excluded.

The primary outcome was the occurrence of osteoporosis (ICD-9-CM 733.0) or pathologic fracture (ICD-9-CM 733.1) during the follow-up period. These two outcomes were followed up independently. All patients were followed up until the occurrence of the primary outcome; December 31, 2011; or withdrawal from the insurance program.

### Statistical analysis

We compared sex, age, and comorbidity between the HSCT and non-HSCT groups by using standardized differences [24]. A standardized difference value < 0.1 indicates a negligible difference in means between the two groups. The incidence density rates of osteoporosis and fracture, stratified by sex, age, and comorbidity, were calculated in both groups. The incidence density rate (per 1000 person-years) of osteoporosis or fracture was defined as the number of incident osteoporosis or fracture divided by the number of person-years. The number of person-years was calculated by summing the number of days from the index date to the date of endpoint. Furthermore, a multivariate Cox proportional hazards regression analysis was performed for measuring the adjusted HRs and 95% CIs of osteoporosis and fracture; these data were compared between the groups after adjustment for sex, age, comorbidity, and cancer type. We used an extended Cox proportional hazards model with the Lunn-McNeil approach (a modified Cox proportional hazards model that considers competing risks) to evaluate the association between HSCT and the risk of osteoporosis/fracture [25]. Finally, the interactions of HSCT with sex, age, and comorbidity status were further examined by adding their product terms into the full model for the evaluation of both osteoporosis and fracture. All analyses were conducted using the SAS statistical software (version 9.4 for Windows; SAS Institute, Inc., Cary, NC, USA), and two-tailed *P* < 0.05 was considered significant.
